# Reverse translational research of autophagy and metabolism in kidney disease: Oshima Award Address 2018

**DOI:** 10.1007/s10157-019-01717-6

**Published:** 2019-03-02

**Authors:** Tomonori Kimura

**Affiliations:** 1 Reverse Translational Research Project, Center for Rare Disease Research, National Institutes of Biomedical Innovation, Health and Nutrition (NIBIOHN), Osaka, Japan; 2 KAGAMI Project, National Institutes of Biomedical Innovation, Health and Nutrition (NIBIOHN), Osaka, Japan; 3 Department of Nephrology, Osaka University Graduate School of Medicine, Osaka, Japan

**Keywords:** Kidney, Autophagy, Disease, Metabolism, Chiral amino acids, Reverse translational research

## Abstract

The management of chronic kidney disease (CKD) has been a great challenge. Focusing on the difficulty to predict the prognosis of CKD, we initially conducted a series of observational studies, and evaluated the prognostic impacts of cardiac, diabetic, kidney, as well as senescent profiles, on CKD. Aiming to protect tubular inflammatory lesions, we studied the roles of autophagy, a process of auto-degradation for cellular homeostasis, in kidney diseases. After having determined its protective role, the proceedings of our autophagy studies are now revealing the mechanisms whereby autophagy protects kidney; autophagy protects kidney from DNA damage, and oxidative and metabolic stress. These emerging roles of autophagy converged on the concept that quality control of organelles (mitochondria and lysosomes), as well as the regulation of metabolism, are the key to protect kidney from diseases, ranging from CKD, acute kidney injury (AKI) to aging kidney. To broaden the clinical potential of autophagy, some cellular and molecular studies were followed up to identify the specific targets of autophagy. Having encountered the critical roles of metabolism in kidney diseases, we conducted a subset of clinical studies, and found that d-amino acids, the chiral derivatives of l-amino acids, can predict the prognosis of CKD. d-Amino acids, normally present in only trace amounts in humans, would be potential candidates for the biomarkers in CKD. The intersections between clinical and basic research provided us a potential approach for the better kidney management, reconfirming the aspects that the reverse translational study is an excellent method for the kidney research.

## Introduction

Chronic kidney disease (CKD) is a global medical problem with its high prevalence in global population (more than 10% in Japanese population), and is closely associated with high morbidity and mortality [1]. The management of CKD has been a great challenge. First of all, no method is currently available to cure chronically damaged kidney. Patients with end-stage kidney disease necessitate kidney replacement therapy, such as hemodialysis or kidney transplantation. Second, prediction of worsening kidney function is difficult. Third, the early detection of CKD is unsatisfactory. These features of CKD have long been the great obstacles for the nephrologists in clinics.

Focusing on the difficulty to predict the prognosis of CKD, our series of observational studies evaluated the prognostic impacts of cardiac, diabetic, kidney, as well as senescent profiles, on CKD [2–4]. These studies strongly motivated us to shift forwards to the basic research of kidney diseases. We launched autophagy study in kidney with a strong aim to protect kidney.

## Autophagy protects kidney

**Fig. 1:**
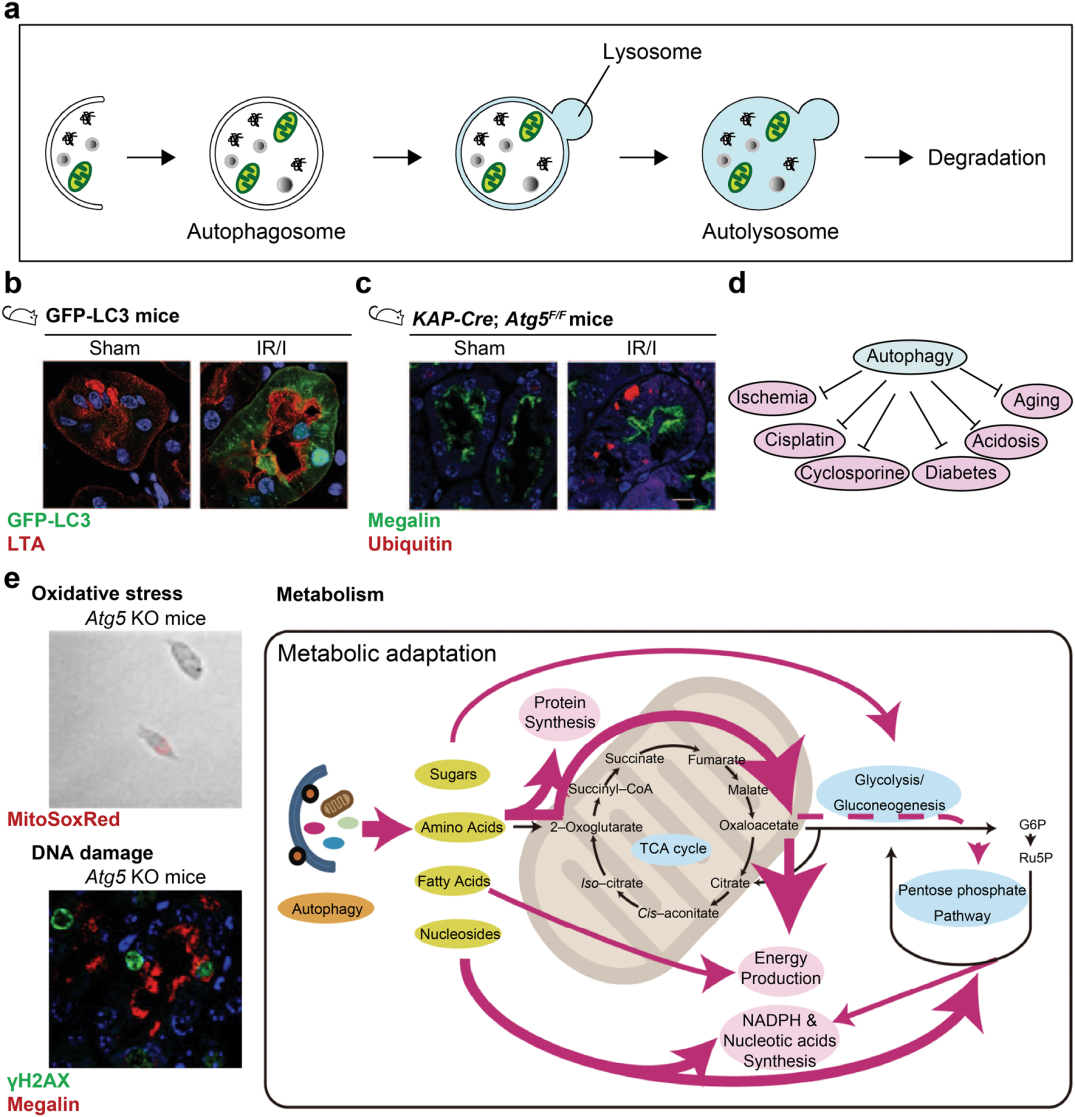
Autophagy protects proximal tubules. **a** The process of autophagy. **b** GFP-LC3 mice showed induction of autophagy, as represented by GFP-positive dots, upon ischemia reperfusion injury (IR/I). Red, *Lotus tetragonolobus* lectin (LTA), a marker for proximal tubules. **c** In proximal tubule-specific autophagy-deficient mice (*KAP*-*Cre; Atg5*^F/F^), IR/I induced the formations of ubiquitin-positive intracellular inclusion bodies. Images were adapted from reference [6] with modification. Green, Megalin, a marker for proximal tubule; red, ubiquitin. **d** Autophagy emerged to be protective against several kidney diseases, injuries, and stresses. **e** Protective mechanism of autophagy against kidney diseases. Autophagy-deficient kidney proximal tubular cells showed accumulation of mitochondria-derived oxidative stress (stained with MitoSoxRed). Autophagy-deficient mice showed accumulation of DNA damage as indicated by γH2AX staining. Thus, autophagy is protective against oxidative stress and DNA damage. Autophagy also plays multiple roles in kidney metabolism. Images were adapted from references [10, 11] with modification

Autophagy is a self-degradation process for cellular homeostasis (Fig. 1a) [5]. Once a part of cellular components, such as damaged mitochondria, is recognized in the cell, autophagosome encloses it, which is followed by fusion with lysosome for degradation. Autophagy is a well-conserved biological process in eukaryotes, and, importantly, has just started to show its protective role in several organs. The process of autophagy is governed by a set of autophagy-related genes (*Atg*). LC3B, a homologue of yeast Atg8, is the component of autophagosome membrane in mammals. Upon induction of autophagy, LC3B proteins are conjugated to a lipid, phosphatidylethanolamine, to mediate the association with the autophagosome membrane.

We examined the role of autophagy in proximal tubules [6]. We focused on proximal tubules of the kidney, since these parts are vulnerable to kidney injuries. Proximal tubules are also characteristics for their highly metabolic state and corresponding consumption of oxygen. For this purpose, we used GFP-LC3 mice [7]. When an autophagosome is formed, GFP-LC3 accumulates to the autophagosome to form GFP dots in GFP-LC3 mice. Therefore, GFP-LC3 dots are used as markers for autophagosome. In these analyses, we found that ischemia reperfusion injury (IR/I) induces autophagy (Fig. 1b).

To elucidate the role of autophagy, we generated proximal tubule-specific autophagy-deficient mice. Since *Atg5* gene, one of *Atg*, is essential for the process of autophagy, *Atg5*-depletion arrests autophagy and is useful for autophagy research. Systemic knockout mice of *Atg5* is not applicable for kidney research, since they are lethal, and tissue-specific autophagy-deficient mice have been used to determine the role of autophagy in tissues [8]. We crossed transgenic mice that expresses Cre recombinase under the control of the promoter of the kidney androgen-regulated protein (*KAP*) gene, which is specifically expressed in proximal tubules, with mice bearing an *Atg5*^flox/flox^ allele, and generated proximal tubule-specific autophagy-deficient mice (*KAP*-*Cre; Atg5*F/F mice) [6]. IR/I induced severer injury in autophagy-deficient mice (Fig. 1c). In addition, IR/I-induced autophagy-deficient mice showed accumulation of ubiquitin-positive inclusion bodies. These findings suggest that autophagy is upregulated by IR/I to degrade ubiquitin-positive abnormal proteins, and that autophagy-deficient resulted in the worsening of kidney due to the failure in the clearance of these abnormal proteins [9].

Subsequently, our studies revealed autophagy protects kidney from several insults, such as cisplatin [10], cyclosporine [11], metabolic acidosis [12], urate crystals [13], as well as stresses from diabetes [14] and aging [6, 15] (Fig. 1d). Mechanistically, autophagy emerged to cover a broad range of defense mechanisms. Autophagy prevents mitochondrial oxidative stress, DNA damages [10], and also controls intracellular metabolism [11, 14] (Fig. 1e). These series of studies have proved a concept that autophagy is applicable for the treatment of a wide range of kidney diseases [16].

## Precision autophagy: a key for clinical application of autophagy

Then, how can we apply autophagy for therapy? We are now planning to regulate inflammation of kidney by autophagy [17]. Autophagy has multiple roles in inflammatory processes, such inflammasome and type I interferon responses that have causal relationships with several kidney diseases. Regulation of inflammation is the key for kidney therapy. The problem here is that autophagy has been considered as a bulk, non-selective process [18]. For therapy, we need to eliminate only what we need to eliminate. These basic ideas led us to explore the precise ways of autophagy regulation [17].

**Fig. 2:**
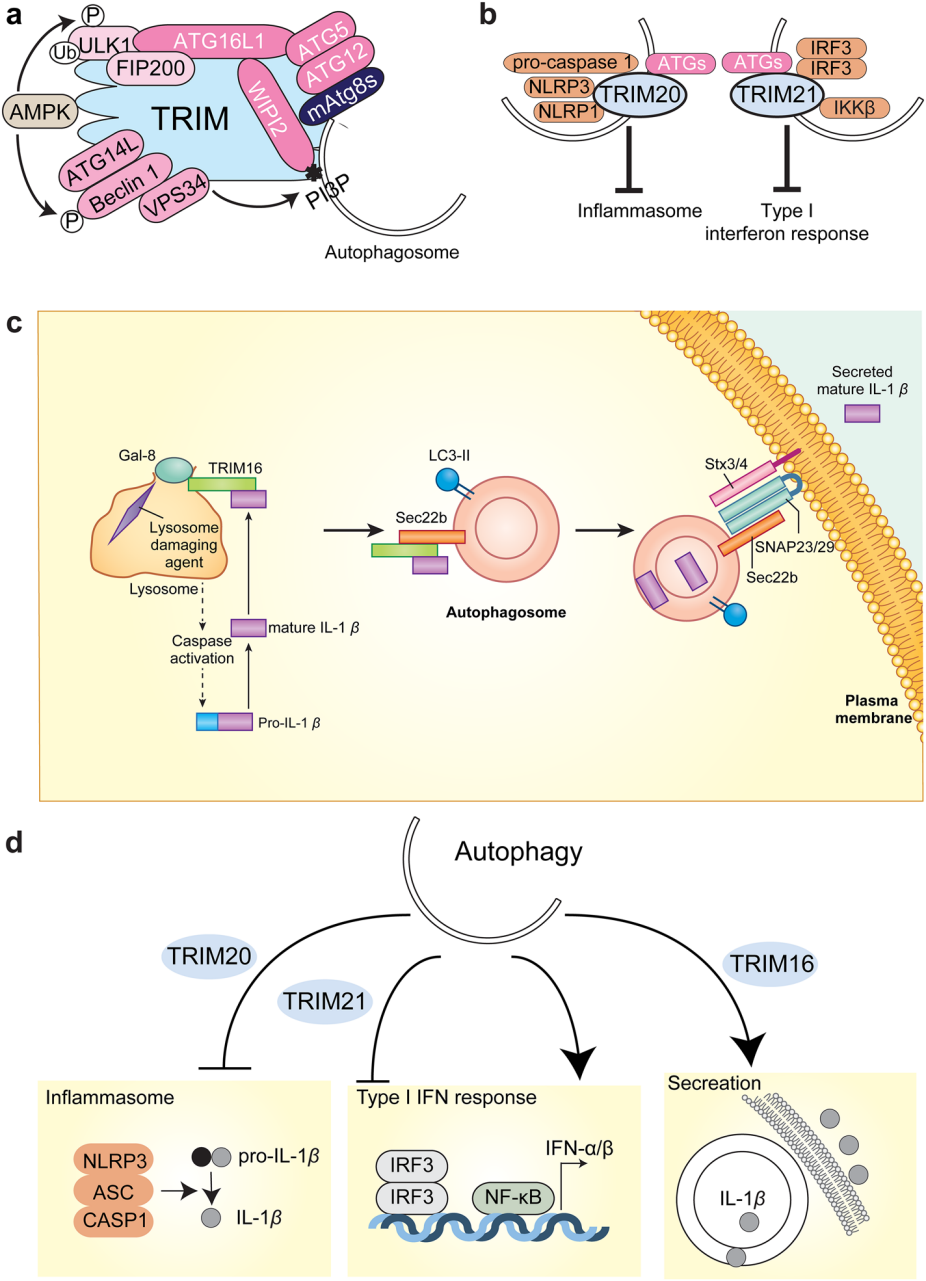
Autophagy for therapy. **a** Molecular platform for precision autophagy. Receptors for precision autophagy (TRIM family proteins as examples) orchestrate both recruitments and activations of autophagy machinery. Depicted are the Atg gene products or associated molecules. **b** Examples of precision autophagy. TRIM20 recognizes its degradative targets and inflammasome components (NLRP3, NLRP1, and pro-caspase 1) to regulate immune responses. Similarly, TRIM21 recognizes signal molecules for type I interferon response (IRF3 and IKKβ). **c** Mode of secretion mediated by autophagy. Autophagy is now emerged to secrete leaderless proteins such as IL-1β. TRIM16 recognizes lysosomal damage via Galectin-8, while binding mature form of IL-1β. TRIM16 then delivers IL-1β to LC3-positive autophagosome membrane fraction, which is Sec22b positive. In combination with a subset of SNARE complex, Sec22b mediates secretion of autophagosome contents. **d** Autophagy regulates precision autophagy and secretion through the selections of their receptors. Shown is an example in the regulation of innate immune response. Images were adapted from references [19–21] with modification

A series of our cell biological studies converged to conceptualize precision autophagy, a highly selective form of autophagy (Fig. 2 a–d) [19, 20]. In this form of autophagy, some specific targets, for example, components of inflammasome such as NLRP3, NLRP1, and pro-caspase 1, are recognized by autophagy receptors (Fig. 2a, b). The receptors, in turn, recruit autophagy machinery (Atg genes’ product and associated molecules depicted in Fig. 2a) for executing degradation (Fig. 2a). Fortunately, we were able to exemplify some of precision autophagy in immune responses [20] (Fig. 2b). In addition, we identified the molecular machinery whereby autophagy promotes secretion, instead of degradation [21] (Fig. 2c). The secretory phenotype of autophagy, a recently recognized phenomenon, is a new field of research whose associations with diseases have just started to be studied (Fig. 2d).

## Chiral amino acids and prognosis of CKD

While seeking for the potential clinical application of autophagy, we came back to one of the biggest problems in CKD management; How can we predict the prognosis of kidney disease? Since we repeatedly observed the close and critical relationship between kidney and metabolism, we applied metabolomics for the prognostic prediction [22, 23].

**Fig. 3:**
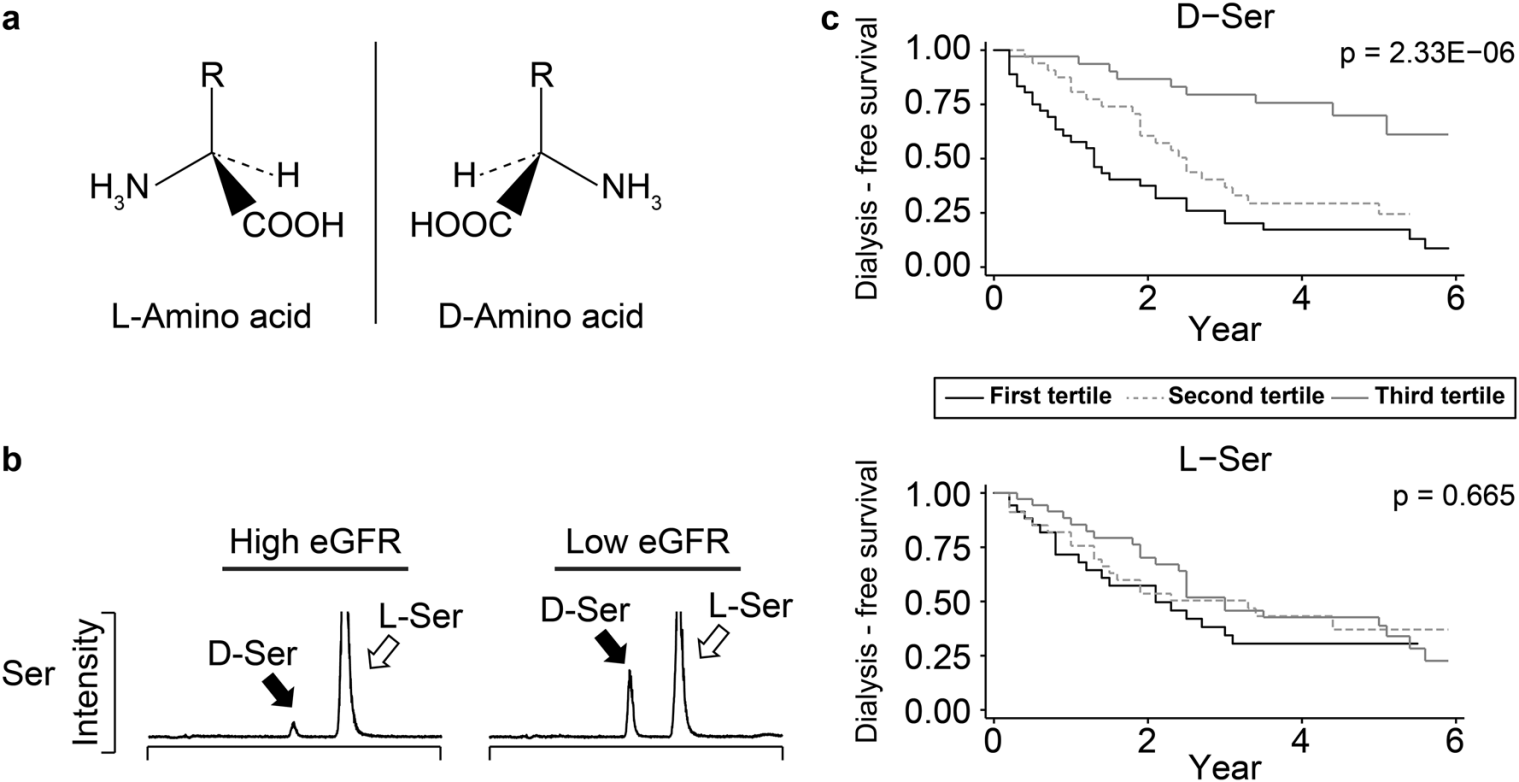
d-Amino acids predict prognosis of chronic kidney disease. **a** Amino acids have chiral centers and consist of two enantiomers, l- and d-amino acids. In nature, only l-amino acids have selectively been detected until recently. **b** Patients with lower levels of eGFR showed higher levels of d-amino acids in plasma. **c** Plasma levels of d-amino acids, but not those of l-forms, predicted the prognoses of CKD patients. Images in **b** and **c** were adapted from reference [22] with modification

For this purpose, we applied chiral amino acid metabolomics [24]. Ever since the discovery of amino acids, scientists have been studying  l-form of amino acids because people only detected l-form in nature (Fig. 3a). Therefore, the presence of  d-amino acids has been overlooked for several decades, until just recently, when the presence of  d-amino acids started to be recognized.

Our recent analyses revealed that the levels of _D_-amino acids were associated with the prognosis of CKD [22]. Trace amounts of  d-amino acids were detected from the plasma of CKD patients, whereas relatively higher levels of d-amino acids were detected from those of advanced stages of CKD patients (Fig. 3b). Higher levels of blood  d-amino acids were associated with the worsening of CKD prognoses (Fig. 3c). Hereby, the potentials of chiral amino acids as biomarkers of kidney diseases are suggested [25].

## Conclusion remarks

In my career as a researcher, I have undergone both clinical and basic studies based on my clinical experience. Clinical and basic studies are an indispensable pair for the reverse translational study, which we nephrologists should pursue. In my case, I started from clinical studies and then shifted forwards to autophagy studies that further led us to the fields of metabolism, immunology, as far as chiral metabolism. The metabolic studies, in turn, invited us to conduct other clinical studies.

Reverse translational research, which solves clinical questions using basic research techniques, is a very powerful approach for kidney diseases, one of intractable diseases. We will keep conducting reverse translational research that goes back and forth between clinical and basic research over CKD, with a final aim for therapy.
